# Effect of boiling on the antidiabetic property of enzyme treated sheep milk casein

**DOI:** 10.14202/vetworld.2016.1152-1156

**Published:** 2016-10-28

**Authors:** Farhath Jan, Santosh Kumar, Richa Jha

**Affiliations:** 1 Department of Biochemistry, Dolphin PG Institute of Biomedical & Natural Sciences, Dehradun, Uttarakhand, India; 2 Department of Biotechnology, Uttranchal University, Dehradun, Uttarakhand, India

**Keywords:** α-amylase, antidiabetic, bioactive peptides, casein, hydrolysates, proteases

## Abstract

**Aim:**

Sheep milk-born bioactive peptides have been found to exhibit various physiological activities. The present work was designed with the aim to evaluate the effect of boiling on antidiabetic property of peptides derived from sheep milk caseinate on hydrolysis with three different proteases.

**Materials and Methods:**

In this investigation, casein prepared from raw and boiled sheep milk was hydrolyzed by three commercially available proteases (trypsin, pepsin, and chymotrypsin). These hydrolysates collected at different hydrolysis times (60, 120, 160, and 240 min) were assayed for their antidiabetic activity.

**Results:**

Among the three different enzyme hydrolysates, casein treated with chymotrypsin shows the highest antidiabetic activity among other enzymes. While the antidiabetic effect of raw milk-derived casein hydrolysates always exceeds than effect shown by boiled milk casein hydrolysates.

**Conclusion:**

The result obtained hence shows that the effect of boiling on the properties of bioactive peptides released during different enzyme digestion depends largely on the enzymatic formulation used and treatment conditions. Chymotrypsin treatment of raw casein yields peptides with maximum antidiabetic activity as compared to pepsin and trypsin. Moreover, the peptides produced after enzymatic treatment of boiled casein show reduced antidiabetic properties. Therefore, enzymatically treated raw milk casein hydrolysates may be used as effective nutritional supplements for diabetic patients, as it causes a significant inhibition of α-amylase activity.

## Introduction

Milk protein in recent times has been recognized to exhibit numerous functionalities *in vivo* by the presence of various bioactive peptides [1]. Milk-born bioactive peptides have been found to exhibit diversified biochemical and physiological activities such as opioid, immunomodulatory, antimicrobial, antioxidative, antithrombotic, cytomodulatory, antihypertensive, and antidiabetic [2]. Antioxidative and antihypertensive property of yak milk casein-derived bioactive peptides has been well reported in recent studies [3,4]. Cow casein hydrolysates produced through hydrolysis with three different protease preparations (pepsin, trypsin, and chymotrypsin), under conditions simulating human digestive tract, exhibits significant antioxidant activity [5]. Among them, the antihypertensive and antidiabetic peptides are of particular interest with respect to present day lifestyle disorders.

Diabetes is one of the major health concerns among the various metabolic disorders, and its incidence is increasing throughout the world. According to a recent report by the WHO on diabetes, about 3% of the world population have diabetes, and the prevalence is expected to double by the year 2025 [6]. Managing diabetes without any significant side effects is still the major challenge faced by the present day medical community. In this, context comes the importance of natural remedial measures like the use of food-derived bioactive peptides. The antidiabetic peptide has also been reported in case of egg yolk protein hydrolysates [7]. These food-borne bioactive peptides can be produced by various methods like processing of food using various physical and chemical agents that hydrolyze proteins, treatment with various proteolytic enzymes, and by the activity of various microbes in case of fermented foods [8]. There are already varieties of interesting applications of milk protein casein-derived bioactive peptides in the form of dietary supplements and as pharmaceutical preparations in the present world. The potential health benefits of sheep milk protein-derived peptides have been a subject of growing research as well as commercial interest in the area of health-promoting functional foods. Hence, these peptides must be incorporated in the form of major ingredients of nutraceutical supplements, dietary formulations, and even in pharmaceutical preparations with the purpose of fulfilling the specific health needs of individuals suffering from specific metabolic disorder

In the current study, three different commercially available proteases were employed in the hydrolysis of sodium caseinate obtained from sheep milk. The biological activities (antidiabetic) of the protein hydrolysates were then assessed, particularly considering the effect of the incubation period and boiling of milk, with the aim of evaluating its potential for applications in food formulations and dietary supplements.

## Materials and Methods

### Ethical approval

Ethical approval is not required to pursue this type of study. However, milk samples were collected as per standard milk collection procedure without giving any stress or harm to animals.

### Extraction of casein

Sheep milk was collected from available breeds of sheep in the locality of Nanda Ki Chowki from Nakli Ram Fields Nursery, Sudhowala, Dehradun. Casein was prepared from sheep milk (both from raw as well as boiled milk samples) using isoelectric precipitation method. Immediately after collection, the milk sample was defatted by centrifugation twice at 5000 rpm for 20 min at 40°C in a refrigerated centrifuge. The filtrate was diluted with equal amount of double distilled water, pH adjusted to 4.6 with 1 N HCL, and finally, the mixture was stirred for 20 min. The precipitate formed was separated by filtration through four layers of cheesecloth, washed, solubilized again in distilled water (equal to the initial volume of milk), and pH was adjusted to 7.0 with 1 N NaOH. The resulting solution was again precipitated with 1 N HCL, and finally, precipitate formed was washed 3-4 times with double distilled water. The same procedure was carried out for boiled milk sample to prepare the casein. The wet caseins after thorough washing with double distilled water kept in cheese cloth and pressed to remove water as much as possible. Then, solid mass of protein formed was air dried at room temperature till it becomes powdery. The concentration of protein in caseins formed was quantified by Lowry’s method of protein determination [9].

### Hydrolysis of casein

Casein prepared was treated with three different enzymes according to the method of Abubakar *et al*. [10] and Pihlanto-Leppala [11] with some modifications (enzyme:substrate ratio is taken as 1:100). All the casein samples were incubated at 37°C for 60, 120, 180, and 240 min, respectively, with their respective enzyme solutions (Table-1). The degree of hydrolysis (DH) by various enzymes was estimated using Hull’s method [12].

**Table-1:** Conditions employed for hydrolysis of sheep milk casein.

Enzyme	Buffer	pH	E/S (w/w)	Temp (°C)
Chymotrypsin	0.02 M ammonium acetate	8.0	1:100	25
Trypsin	0.05 M Tris HCL	2.0	1:100	37
Pepsin	0.05 M HCL	2.0	1:100	37

### Assay of antidiabetic activity

Antidiabetic activity was measured by α-amylase inhibition of the casein hydrolysates as per the modified method of Sigma-Aldrich [13]. Each sample assay is carried out in triplicate, and data were represented as a mean value along with the standard deviation.

## Results

**Figure-1:**
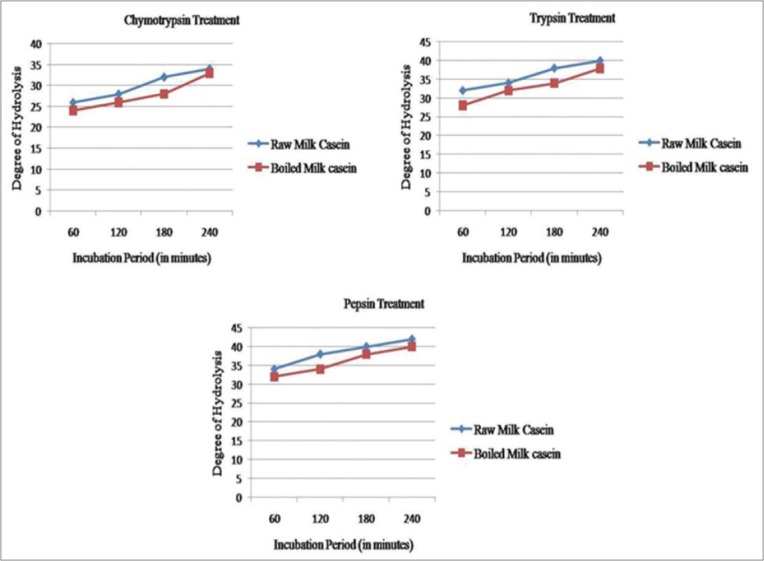
Degree of hydrolysis with different enzymes.

The dry weight of casein was found to be 3.26 g/100 ml of sheep raw milk and 5.17 g/100 ml of sheep boiled milk. The total protein content was found to be 400 µg/10 µl and 370 µg/µl of sodium caseinate, in case of raw and boiled milk caseins, respectively. DH of casein samples increases with the increase in incubation time reaching maximum at 4 h, in case of all enzymes (Table-2). Trypsin and pepsin treated raw casein shows maximum DH than boiled casein. Further, the DH of raw casein with pepsin treatment was significantly higher than on treatment with trypsin and chymotrypsin (Figure-1); this suggests that the enzymes could not further hydrolyze the remaining bonds within the generated peptides (as this depends on the by enzyme specificity) and pepsin utilized more protein as its substrate to cause greater DH. Chymotrypsin treatment yields minimum DH indicating that sheep casein is more resistant to this enzyme.

**Table-2:** DH of sheep casein by different proteases.

Incubation period (in min)	% Hydrolysis					

	Chymotrypsin		Trypsin		Pepsin	
		
	Raw	Boiled	Raw	Boiled	Raw	Boiled
60	26	24	32	28	34	32
120	28	26	34	32	38	34
180	32	28	38	34	40	38
240	34	33	40	38	42	40

DH=Degree of hydrolysis

**Figure-2:**
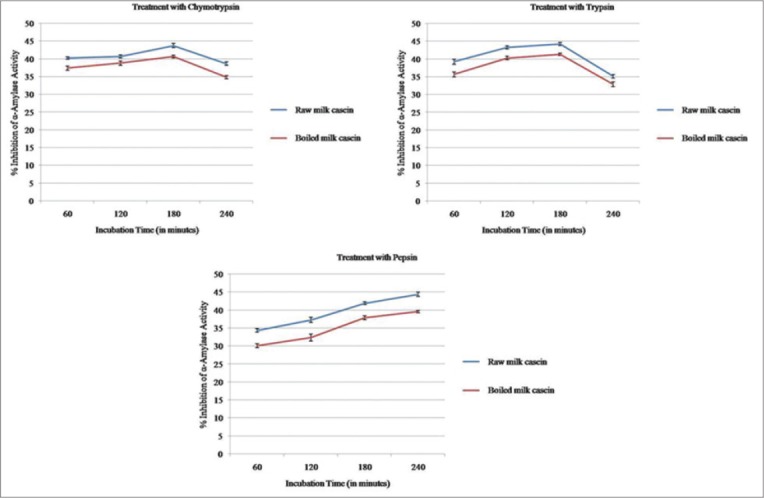
Antidiabetic assay different enzyme hydrolysates.

The comparative inference of antidiabetic status of hydrolysates (raw and boiled) with reference to different incubation periods was tabulated (Table-3). Among the three different enzymes, hydrolysates treated with chymotrypsin shows highest antidiabetic activity than hydrolysates obtained with trypsin and pepsin enzymes. Overall, raw milk casein hydrolysates always showed the higher antidiabetic effect as compared to boiled milk casein hydrolysates, irrespective of the enzyme used (Figure-2).

**Table-3:** Antidiabetic status of sheep casein hydrolysates.

Incubation period (in min)	% Inhibition of α-amylase activity					

	Chymotrypsin hydrolysates		Trypsin hydrolysates		Pepsin hydrolysates	
		
	Raw	Boiled	Raw	Boiled	Raw	Boiled
60	40.26±0.30	37.38±0.59	39.25±0.67	35.67±0.66	34.37±0.44	30.11±0.54
120	40.70±0.44	38.79±0.58	43.29±0.39	40.30±0.51	37.30±0.73	32.35±0.96
180	43.77±0.59	40.65±0.36	44.26±0.44	41.33±0.36	41.94±0.38	37.87±0.51
240	38.69±0.44	34.84±0.44	35.16±0.51	32.89±0.65	44.38±0.59	39.62±0.30

## Discussion

Casein prepared isoelectrically was treated with three different enzymes so as to break the caseinates into peptides having desired biological activities. These peptides obtained after hydrolysis were further analyzed for extend of hydrolysis. The DH was estimated by quantifying the undigested protein as well as measuring the content of peptide bonds cleaved during different enzymatic treatments. The increase in the released amino acids relates to the increase in the DH. The decrease in the DH after boiling may be due to the denaturation of milk casein due to which the respective enzymes fails to recognize their specific binding sites and thereby unable to cleave its specific structural motifs. Denaturation hampers enzyme specificity reducing the peptide generation, which ultimately lead to the reduction in antidiabetic properties of bioactive peptides. Therefore, the biological activity of peptides generated depends mainly on the substrate, specificity of enzymes used and hydrolysis conditions used [14-16]. The DH usually increases with the increase in incubation time which correlates with the reduction in bioactive properties of peptides.

Further, suppression of the activity of intestinal digestive enzymes (α-amylase and α-glucosidase both of which are important enzymes involved in digestion of carbohydrates and subsequent absorption of glucose in our body) would delay the hydrolysis of various complex carbohydrates and thereby reduce the availability of glucose in blood, which would, in turn, retard glucose absorption in digestive organs and ultimately resulting in the reduction of postprandial blood glucose level in diabetes [17].

It has already been reported that antidiabetic effect of milk casein is primarily due to its composition of bioactive peptides which after release during *in vivo* enzymatic digestion, causes the secretion of gut-derived hormones and/or inhibition of enzymes involved in glucose metabolism [18]. Suppression of these enzymes leads to delay in carbohydrate digestion which, in turn, increases overall digestion time of carbohydrates. Thereby, glucose is less absorbed due to delayed carbohydrates hydrolysis into glucose, and hence, the postprandial blood glucose level and insulin level diminishes [19].

## Conclusion

Our findings revealed that casein hydrolysates generated by different enzymatic treatments show significant antidiabetic properties. The role of such peptides as antidiabetics was revealed and it was observed that chymotrypsin treatment of raw casein yields peptides with maximum antidiabetic activity as compared to those formed by pepsin and trypsin treatment. Moreover, the peptides produced after enzymatic treatment of boiled casein show reduced antidiabetic properties. It means this property is adversely influenced by boiling. Increase in the temperature denatures milk proteins and reduces the enzymatic specific and activity, leading to significant decrease in the peptide content. Thus, boiling indirectly and adversely influences the formation of bioactive antidiabetic peptides. It can be concluded that bioactive peptides derived from chymotrypsin and pepsin treatment of raw milk exhibiting maximum antidiabetic activity, could be used as a nutritional supplement for diabetic patients. Moreover, sheep casein peptides generated by enzymatic treatments exhibiting a significant effect on lowering blood glucose level may be further explored to sequence and identify the individual peptides involved in imparting antidiabetic activity. Further *in-vitro* studies are essential to explore the commercial importance of these bioactive peptides as nutritional formulations for diabetic patients.

## Authors’ Contributions

FJ is master’s student, who performed the research work under the guidance of SK. Dr. RJ was the co-guide involved in the manuscript preparation. All authors read and approved the final manuscript.
